# Improved Mechanical Performance in FDM Cellular Frame Structures through Partial Incorporation of Faces

**DOI:** 10.3390/polym16101340

**Published:** 2024-05-09

**Authors:** Mahan Ghosh, Nandika Anne D’Souza

**Affiliations:** 1 Mechanical Engineering, University of North Texas, 1155 Union Circle #310440, Denton, TX 76203-5017, USA; mahanghosh@my.unt.edu; 2 Materials Science and Engineering, University of North Texas, 1155 Union Circle #310440, Denton, TX 76203-5017, USA

**Keywords:** FDM, compressive testing, BCC lattice, strut, face, moment of inertia

## Abstract

The utilization of lattice-type cellular architectures has seen a significant increase, owing to their predictable shape and the ability to fabricate templated porous materials through low-cost 3D-printing methods. Frames based on atomic lattice structures such as face-centered cubic (FCC), body-centered cubic (BCC), or simple cubic (SC) have been utilized. In FDM, the mechanical performance has been impeded by stress concentration at the nodes and melt-solidification interfaces arising from layer-by-layer deposition. Adding plates to the frames has resulted in improvements with a concurrent increase in weight and hot-pocket-induced dimensional impact in the closed cells formed. In this paper, we explore compressive performance from the partial addition of plates to the frames of a SC-BCC lattice. Compression testing of both single unit cells and 4 × 4 × 4 lattices in all three axial directions is conducted to examine stress transfer to the nearest neighbor and assess scale-up stress transfer. Our findings reveal that hybrid lattice structure unit cells exhibit significantly improved modulus in the range of 125% to 393%, specific modulus in the range of 13% to 120%, and energy absorption in the range of 17% to 395% over the open lattice. The scaled-up lattice modulus increased by 8% to 400%, specific modulus by 2% to 107%, and energy absorption by 37% to 553% over the lattice frame. Parameters that emerged as key to improved lightweighting.

## 1. Introduction

Lightweighting structures are vital for fuel economy in aerospace, automotive, and other transportation applications as well as in conveying products from one point to another. Porosity has been a strategic method of increasing strength-to-weight and modulus-to-weight ratios. Polymer foams are, therefore, a high-volume product used in applications as diverse as cushions, food trays, cups, and structural cores in sandwich composites. Multifunctional performance, such as thermal insulation, acoustic absorption, lightweighting, energy absorption, and impact performance, is enabled through porous architectures. The mechanical performance of porous structures has been analyzed by Gibson and Ashby [1]. They describe a cellular solid as one made of an interconnected network of solid struts (open cell) [2,3,4] or plates that form the edges of faces of cells (closed cell) [5]. Three topographies characterize cellular structures formed from plates and struts. One is ordered prismatic polygon columns, such as hexagonal honeycomb sandwich cores, [1,6], the second is open pores from the frames, and the third is closed pores with walls over the frames. In the case of open- and closed-celled foams, formed from chemical/physical foaming, the stochastic nature of cell size, cell shape, and wall thickness makes the attribution of cause and effect on performance challenging. Even naming foams as open and closed cells is subjective due to the variation across a foam. This has led to the use of density ratios. For example, soft polymer foams that are open cells have density ratios around 0.05 while rigid foams have ratios around 0.2. Increased density ratios are accompanied by more faces, wall thickness, and closed cells. When the density of the foam is more than 30% of the bulk material, the material begins to have a less porous structure. In our recent work with foaming polylactic acid with microcellulose using carbon dioxide [7], the thermal conductivity in the cellular foams was significantly impacted by cell wall thickness while the compression modulus was affected by the intrinsic properties of the PLA and extent of open porosity. The attribution of performance to geometry, cell density, and cell wall thickness in stochastic foams is therefore difficult. Thus, regularly ordered porous structures are advantageous. The advent of 3D printing has led to more exploration of the architectures of cellular materials. Honeycombs and other columnar systems are transitioning from non-prismatic cellular structures to periodic lattice structures. Periodic lattices describe structures formed by repeating unit cells designed in three orthogonal directions. The unit cell is an interconnected network of ligaments or struts [1,8]. The mechanical behavior of the interconnected network is affected by the architecture of the repeating unit and is ascribed to the connectivity described by the Maxwell criterion [9]. In three dimensions, the criterion [10] is described by Equation (1) where *M* indicates the difference between the redundant struts (beyond that required for the joint to be statically determinate), *r* represents the state of self-stress, *m* is the number of extension free mechanisms in the lattice, *s* is the number of struts, *j* is the number of joints, and *k* is the number of kinematic constraints or fixed motion of joints, which would be six for a 3D truss.

(1) M=r−m=s−3j+k

Two responses were considered. Ashby determined that struts would respond either to bending or to axial stretch deformation based on the constraints imposed on the joints or r − m. If a joint was unrestrained in any degree of freedom, the lack of resistance would affect the deformation mode of the connected structure, and stretching would ensue. Constraints to displacement introduced by the addition of struts would induce bending deformation. When the number of struts is greater than that needed for static determinacy, M would be >0, and the lattice deforms through an axial stretching response. In contrast, when the struts were lower than that required for static determinacy, a bending response would ensue. Ashby predicted that a lattice that deformed in stretching response would yield three times the stiffness of the lattice that responded in bending. Stretch efficiency would make stretch-governed structures more effective for increased modulus-to-density and strength-to-weight considerations. Deshpande, Ashby, and Fleck [11] predicted that cells that are comprised of plates would carry membrane stresses that buckle or rupture at stresses so low that their contribution would not change the scaling laws since the bulk of the load would be carried by the cell edges. In both cases of frames or plate architectures, the Ashby inferences are based on assumptions that the nodes are pin joints and the frames carry only membrane stresses. Further loading direction is assumed to not have an effect. Ashby and Gibson proposed the following equations for distinguishing between stretch- and bend-dominated structures.

(2) $$E^{*}=CE_{s}{\left(\frac{\rho^{*}}{\rho_{s}}\right)}^{n}$$

(3) $$\sigma_{pl}^{*}=C{\sigma_{y}}_{,}s{\left(\frac{\rho^{*}}{\rho_{s}}\right)}^{m}$$

(4) $$\sigma_{cl}^{*}=C\sigma_{s}{\left(\frac{\rho^{*}}{\rho_{s}}\right)}^{p}$$

Here, $E^{*}$ is the modulus of the lattice, and $E_{s}$ is the modulus of the material. $\rho^{*}$ is the density of the lattice, and $\rho_{s}$ is the density of the material. $\sigma_{pl}^{*}$ is stress on lattice at plastic collapse, and ${\sigma_{y}}_{,}s$ is the yield stress of the material. $\sigma_{cl}^{*}$ is the stress in the lattice at elastic collapse, and $\sigma_{s}$ is the stress in the solid. When *n* = 2, *m* = 1.5, and *p* = 2, the structure is bend-dominated. When *n* = 1, *m* = 1, and *p* = 2, the structure is stretch-dominated.

The mechanical performance of numerous lattice architectures has been explored by researchers. High specific strength, modulus, impact resistance, and energy absorption capacity have been described in transforming brittle ceramics [12,13]. The simple cubic (SC) architecture of “boxes” has been utilized as all struts, all plates, and hollow struts filled with materials of varying moduli with arrangement to produce isotropic performance [14]. The body-centered cubic (BCC) and the face-centered cubic (FCC) have also been extensively investigated [15,16,17,18]. The FCC lattice, particularly due to the 12 struts emanating from a single node, has been further investigated as octet and tetrahedral lattices. When the FCC and BCC lattices are paired to the simple cubic frame, there is a significant increase in mechanical modulus and stress arising from the addition of struts at the same joint. These are termed SC-FCC or FCCZ, and SC-BCC or BCCZ. There has also been research in reducing the stress concentration on the nodes of a BCC lattice by increasing the size of the nodes of the struts at the junctions with minimal increase in weight, see Liu et al. [19]. They achieved an improvement in energy absorption, elastic modulus, and yield strength by 11.89%, 61.80%, and 53.72%, respectively. An FCC lattice was modified by Wang et al. by the addition of two struts at the center of the lattice. This design resulted in an improvement of 49.3% in energy absorption under high-impact loading by changing the deformation mode [20]. There have also been efforts to create structures using topology optimization. Li et al. used an FCC lattice structure as the base cell and created a bigger structure and used topology optimization to increase the strut diameters at localized regions based on the loading condition and demonstrated increased rigidity based on the application [21]. These cases demonstrated the fact that by strategically adding material, we can achieve higher lightweighting compared to just using basic strut-based lattices. 

In addition to lattice geometry, manufacturing with fused deposition modeling (FDM) has had a significant impact on performance. When these lattices are processed using FDM 3D printing, the solidification of the newly deposited layer and sequence of remelting leads to delamination. Further, when nodes are formed, they become a point of stress concentration. Thus, in addition to the architecture of the unit cell, examining multiple cells enables scaling up to structural applications through examination of the effect of nearest neighbor cells. The examination of the architecture goes beyond the FDM method. Delamination and stress concentration are also widespread in other deposition methods as well as for metals and ceramics. Lohmuller et al. [22] modeled the effect, developing a relationship that quantified stress concentration and relative density to qualify the performance of strut lattices. The stress concentration is further amplified by manufacturing defects in 3D printing as reported by Boniotti et al. [23] All-face lattices have also been explored by Berger et al. [24], and the results indicate higher modulus and strength are achieved. They demonstrated that a theoretical limit of strain energy storage in metamaterials was possible only by using plates for constructing these metamaterials. These lattice structures end up having designs that have their internal faces subjected to trapped air and their outer surfaces subjected to outer conditions, which can cause uneven heat distribution during printing, which causes warping and deformation of printed structures and leaves no room for post-processing, if needed [25], thus making them infeasible for practical applications.

Keeping in mind that solidification in all-face lattices and stress concentration in frames are challenges all strut-based frame lattices, the objective of this paper is to examine the potential of the partial introduction of plates in mitigating the stress concentration of open cells and ensuring no closed pockets for air entrapment. To do this we utilize a BCC-SC or BCCZ unit cell and sequentially add faces to the unit cell (Table 1). We use FDM manufacturing with polylactic acid (PLA) filament to print these designs. The modulus, specific modulus, yield strength, specific yield strength, energy absorption, specific energy absorption, and deformation modes are analyzed in our study. The effect of placing the plates to minimize strut buckling is probed by orienting the lattice in the x, y, and z directions. Stress transfer and scale-up in FDM manufacture are a significant concern. Thus, we create both a unit cell and a 4 × 4 × 4 (64-cell) lattice to explore the effect of the lattice and scale-up potential. The 4 × 4 × 4 cell arrangement enabled a 2 × 2 × 2 cube to be contained within a core lattice that enabled mitigation of surface crushing effects experienced during testing.

## 2. Materials and Methods

### 2.1. Material, Lattice Design, and Fabrication

The filament used for the FDM was a PLA. The filament and the cells were examined for extent of crystallinity using Differential Calorimetry (DSC), density and mechanical properties of tension (ASTM D638-14) [26], compression (ASTM D695-15) [27], and flexure (ASTM D790-17) [28]. For crystallinity, struts and plates were examined at over 6 locations. A melting temperature of 176 degrees Celsius and an enthalpy below 63 J/g were obtained. This suggests that our PLA is an alpha-type largely amorphous PLA [29]. The density of the 3D-printed filament was determined using a cube of 10 × 10 × 10 mm^3^ 100% infill. The density of the material was determined to be (1.16 × 10^3^ kg/m^3^).

The mechanical properties determined are shown in Table 2.

Table 1 shows the unit-cell configuration and orientation of the 3D-printed strut-face lattice. The nomenclature employed for sample identification is PLA-A-Q, where A represents the number of faces in the lattice above the midplane of the cube and Q represents the load direction for compression testing. For the lattice having two faces, two options arise with faces adjacent and opposite, as shown in Table 1. When the faces are positioned opposite each other, the nomenclature used is PLA-A-O-Q. 

The 3D printing was conducted along the plane perpendicular to Q. The lattice was analyzed for planes of symmetry and, consequently, 13 unique orientations were identified. For all lattice types, a single cell of 10 mm × 10 mm × 10 mm with strut diameter of 1.5 mm was utilized. This led to the unit cube cell having a dimension of 11.5 mm on all sides. A corresponding set of a 4 × 4 × 4-unit-cell lattice was also printed using the same unit-cell dimensions resulting in a cube of 41.5 × 41.5 × 41.5 mm^3^. Computer-Aided Design models were first created using Autodesk Inventor Professional 2019, then exported in STL format, and then fed into the Ultimaker Cura 2.0 for slicing and creating the G-code. Each strut cross-section was circular. Ender 3 Pro printer by Creality (Shenzen, China) was used to manufacture the samples. The print bed was maintained at 60 °C, and the deposition temperature was at 210 °C. The nozzle diameter is 0.4 mm, and all the samples were printed at 100% density. The printing speed was 50 mm/s, and the layer thickness was maintained at 0.4 mm. 

### 2.2. Lattice Compression Testing

Single unit cells were fabricated and tested on a Shimadzu AGS-X 10 kN machine (Nanjing, China). The 4 × 4 × 4 lattice elements were fabricated and subjected to compressive loading on the 810 MTS 500 kN load capacity (MTS Systems Corp, Eden Prairie, MN, USA). Both tests were conducted at a strain rate of 1 mm/s, up to complete densification. The strain rate was selected to be within the <2 mm/min rate recommended by the ASTM compression testing standard. Three specimens of each sample for compression testing were fabricated and tested. The print direction was kept the same as the direction of loading for each sample to eliminate variations due to the stepping effect of FDM printing. The dimensional accuracy between CAD and the printed lattice was analyzed and determined to be less than 0.1%. The force was converted to stress by dividing by the projection area (11.5^2^ for a single cell and 41.5^2^ for the 4 × 4 × 4 lattice). The strain was calculated by dividing the cross-head displacement by the height, which was 11.5 for the single cell and 41.5 for the 4 × 4 × 4 lattice. The slope of the stress–strain curve was used to calculate the modulus.

### 2.3. Density of the Lattice

The density of PLA was calculated using a 40 mm × 40 mm × 40 mm 3D-printed cube. The weight was determined on a weighing scale manufactured by Fisher Science Education, having the least count of 0.0001 g. The PLA density was determined to be 1.16 × 10^3^ kg/m^3^. To determine the lattice density, the volume was calculated theoretically using Autodesk Inventor, which computes the occupied volume from the CAD rendering. The theoretical lattice density was determined using the weight corresponding to the CAD volume. The experimental density was calculated using the weight of the lattice measured on the weighing scale. The deviation between experimental and theoretical density was found to be no greater than 6%, and thus the theoretical density was used in calculations for specific modulus, yield stress, and energy absorption. Vernier calipers with a least count of 0.1 mm were used to examine the lattice struts and plates, and a 0%-dimensional difference was noted.

### 2.4. Finite Element Analysis Boundary Conditions

The simulation was performed on an Ansys workbench 2020. The CAD files created on Autodesk Inventor Professional 2020 were converted into step files and imported into Ansys. In Ansys, input material properties are required, namely, the modulus, yield point, Poisson’s ratio, and density. The values utilized for the simulations were from the compression test provided in Table 2. The Poisson’s ratio was assumed to be 0.33 [30], and the frictional coefficient was set at 0.1. Tetrahedral fine meshing was employed for the simulation, which resulted in around 45,000 nodes for the unit cell and 2,880,000 nodes for the 4 × 4 × 4 structures. The lattice was placed in between two steel plates keeping the bottom plate fixed. The top plate was displaced 5 mm over 60 s. The reaction force from the bottom plate and the displacement of the top plate were captured by Ansys 19.2 (Canonsburg, PA, USA) with an interval of 0.01 s. The tabulated force and displacement were then converted in a spreadsheet software (Microsoft Excel, Version 2403) to stress and strain. To determine the stress, the reaction force was divided by the projected cross-section of the lattice, which was 11.5 × 11.5 mm^2^ for the unit cell and 41.5 × 41.5 mm^2^ in the case of the 4 × 4 × 4 scaled-up lattice, to obtain the stress in the lattice structure. The displacements were divided by the original distance between the plates, which was 11.5 mm for the unit cell and 41.5 mm for the 4 × 4 × 4 scaled-up lattice. The slope of the stress–strain curve was extracted as the modulus.

## 3. Results and Discussion

### 3.1. Effect of the Addition of Faces on the Unit Cell

**Figure 1:**
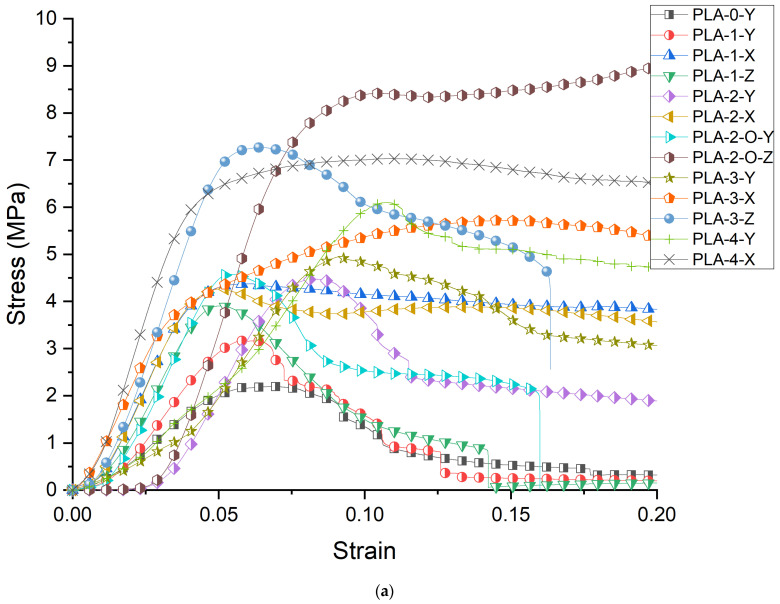

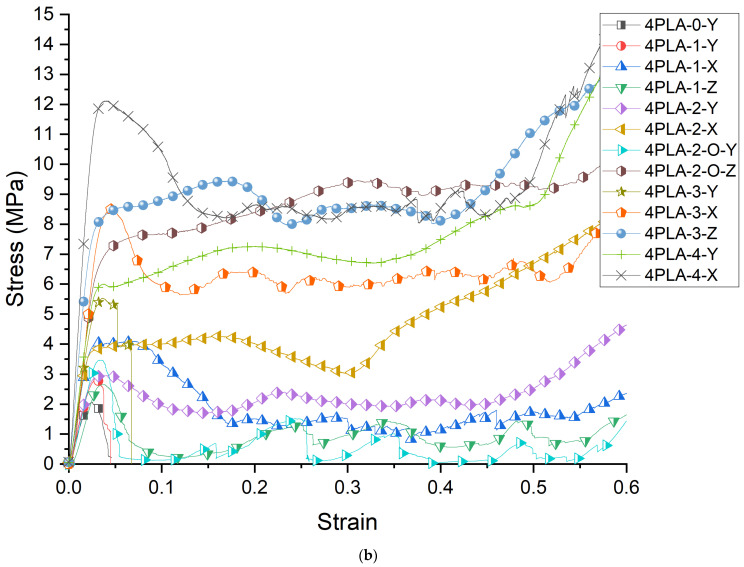
(**a**) Stress–Strain response of the unit cells; (**b**) Stress–strain response of the 4 × 4 × 4 cellular lattice.

**Figure 2:**
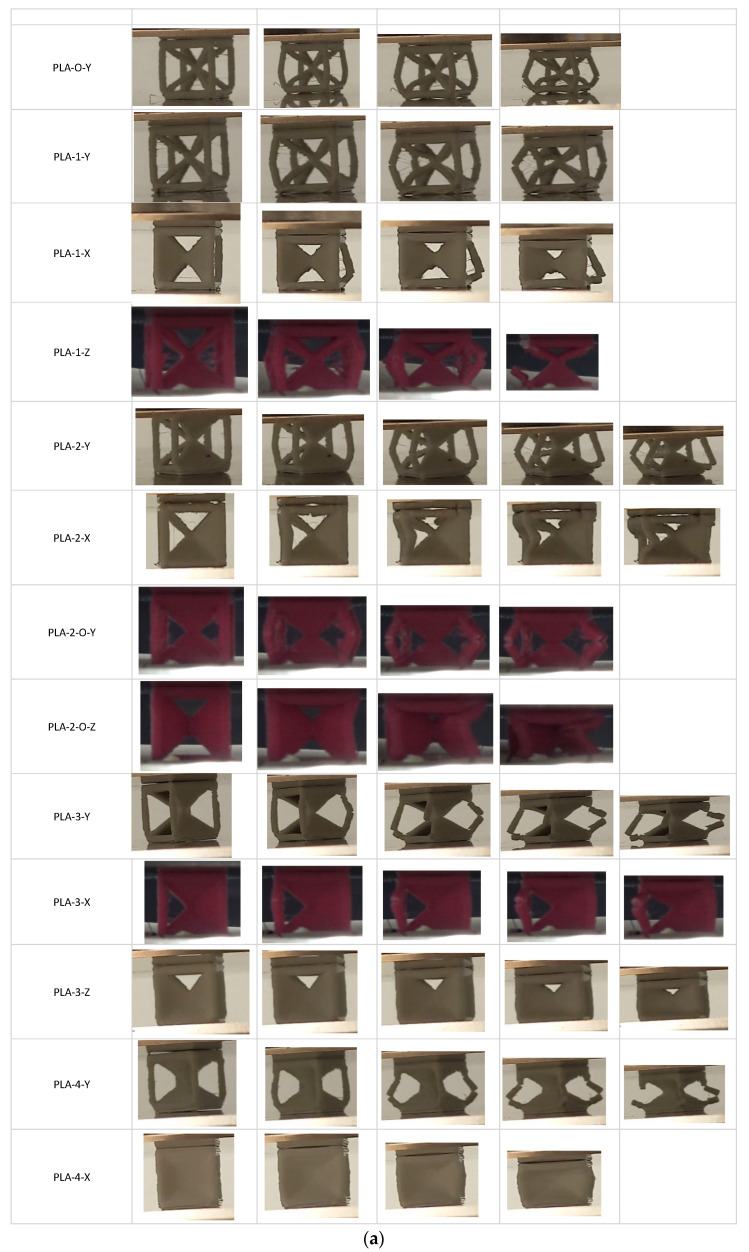

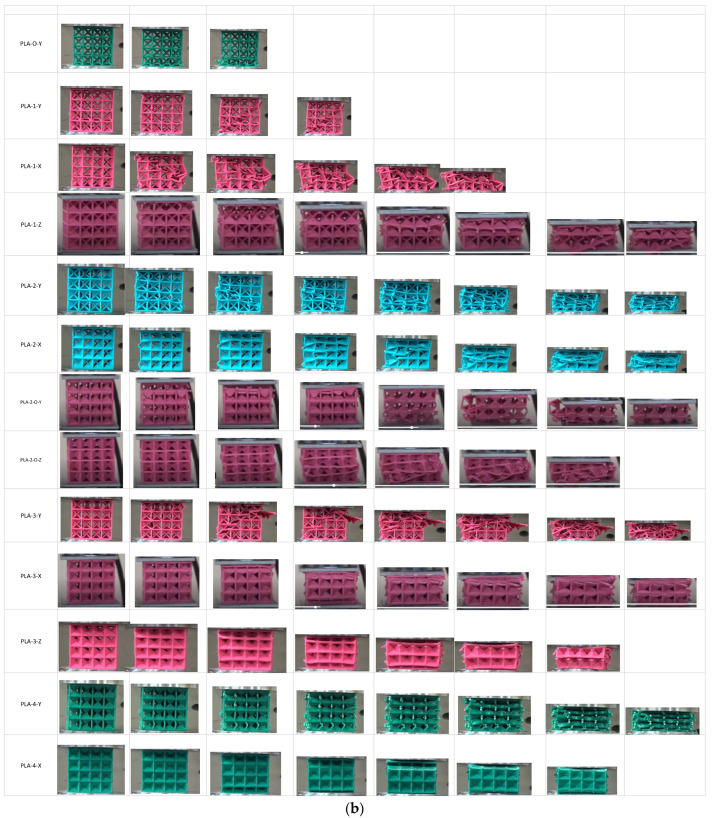
(**a**) Experimental images during compression showing unit-cell fracture; (**b**) Experimental images during compression showing 4 × 4 × 4 lattice fracture.

**Figure 3:**
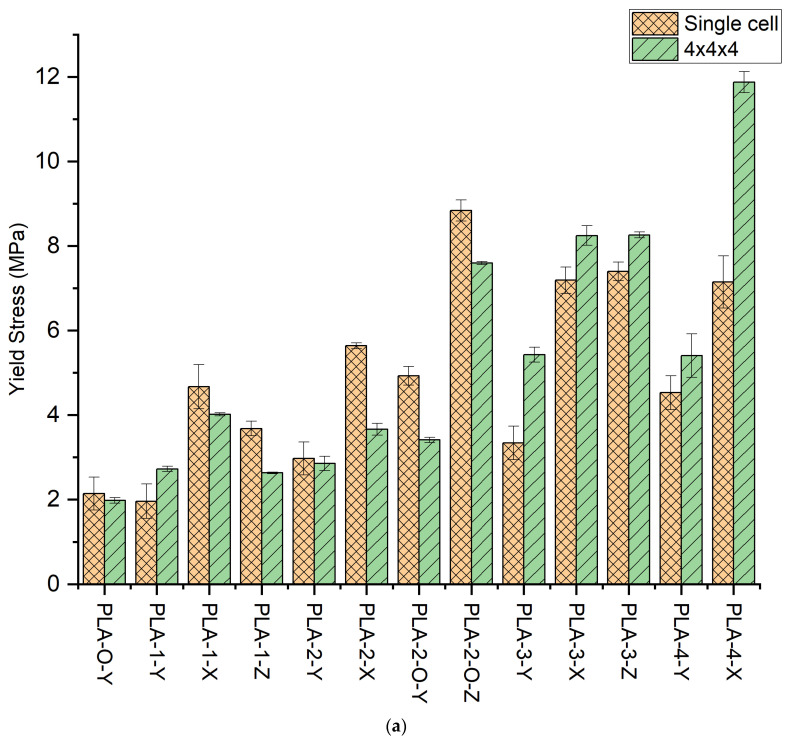

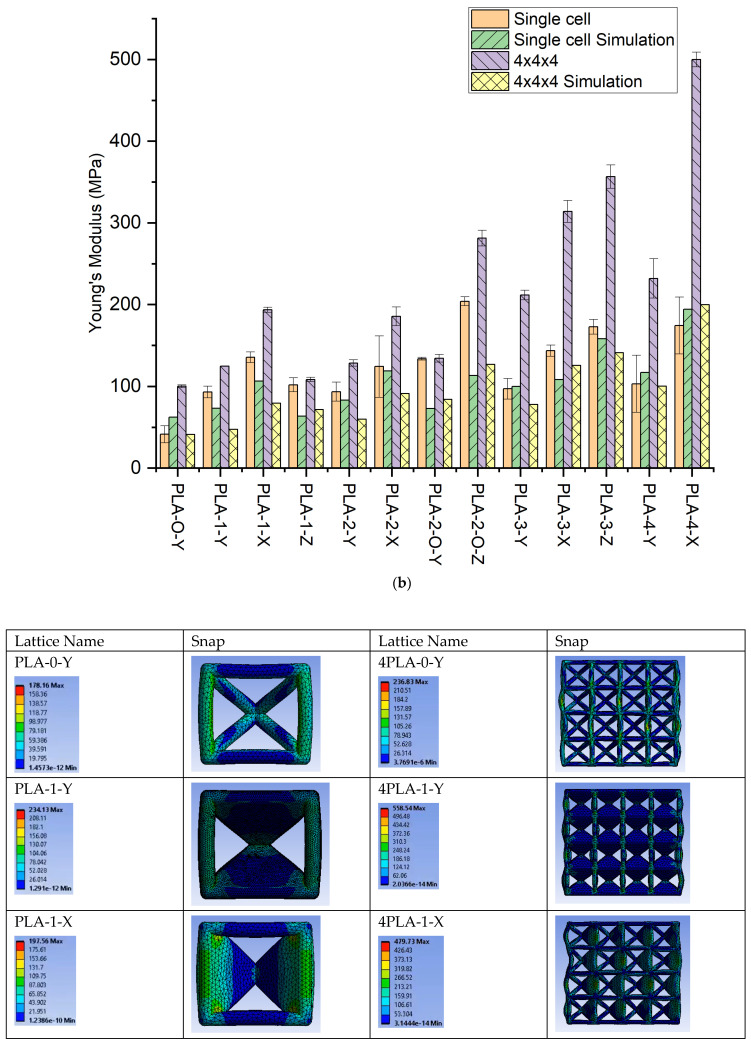

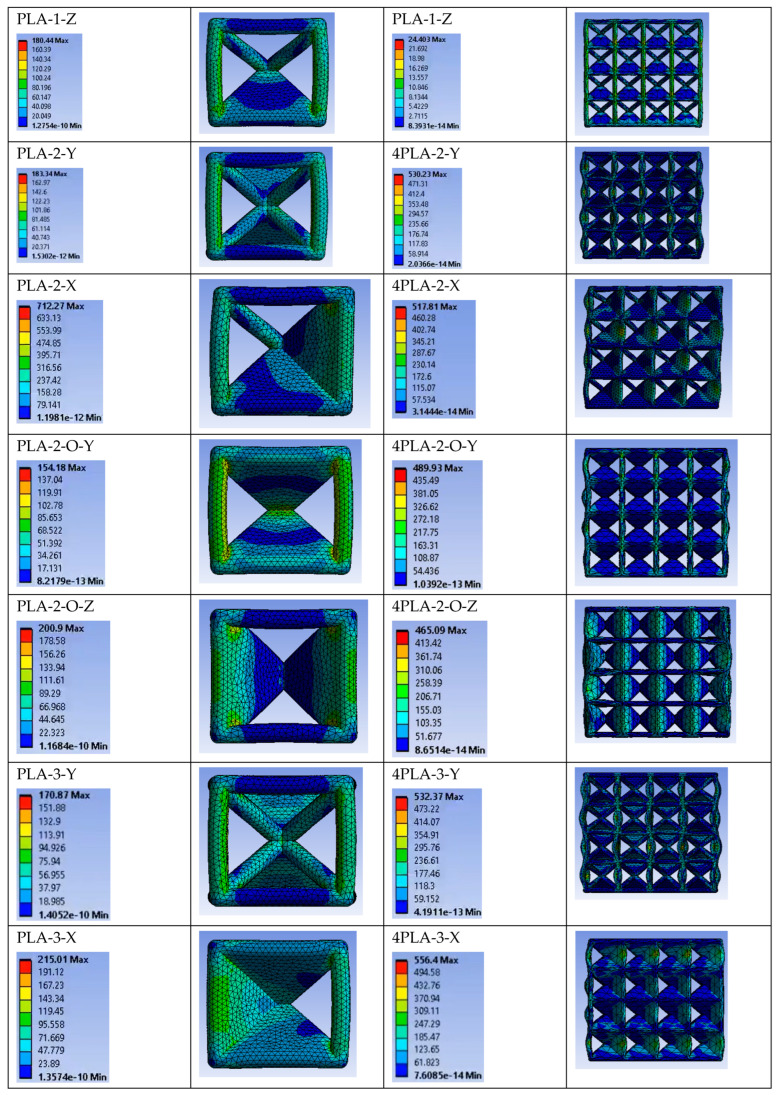

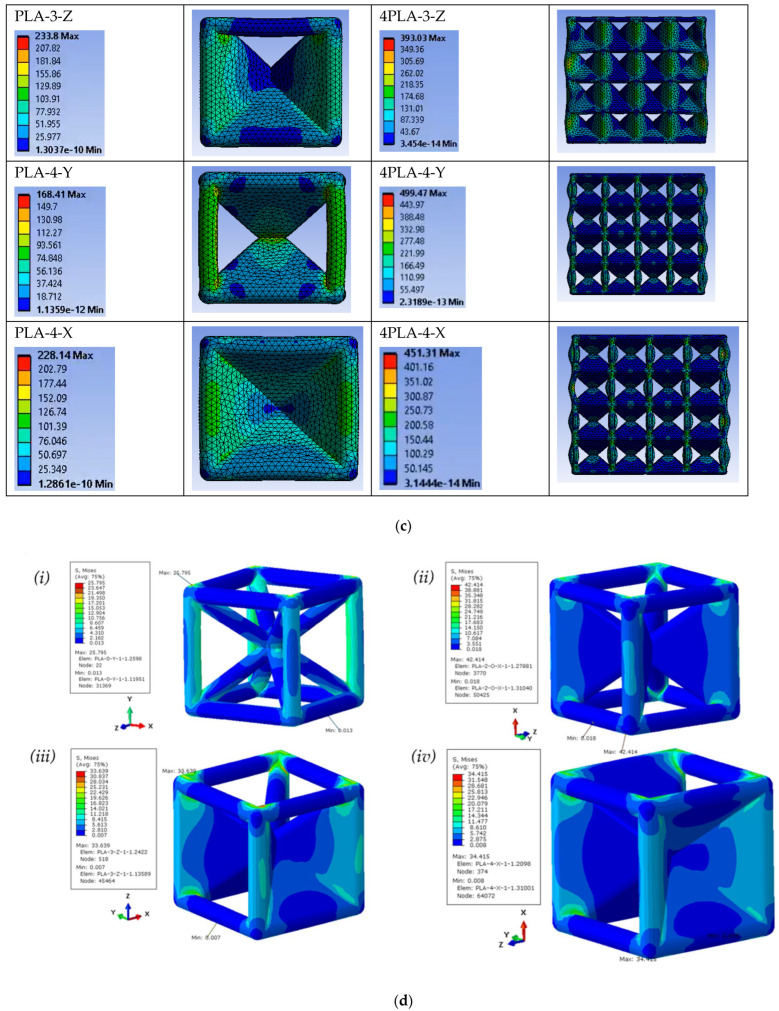
(**a**) Yield stress for single and 4 × 4 lattices for all 13 configurations; (**b**) Elastic Modulus for single and 4 × 4 lattice for all 13 configurations with simulations; (**c**) Stress contours in unit cell and 4 × 4 × 4 simulations of all 13 lattice types; (**d**) Stress contours in unit-cell simulations of (i) PLA-0-Y, (ii) PLA-2-O-Z, (iii) PLA-3-Z, and (iv) PLA-4-X at a displacement strain of 0.1 mm.

The stress–strain curves for the unit cells for all 13 configurations are shown in Figure 1a. The curves reflect the shape typical of bulk materials with an initial linear elastic regime followed by strain softening or strain hardening. We first examine the effect of placing faces in the Y direction. Comparing PLA-0-Y, PLA-1-Y, PLA-2-Y, PLA-2-O-Y, PLA-3-Y, and PLA-4-Y, the curves show a similar strain-softening response except for the four-face lattice. For PLA-4-Y, following an initial peak and drop in stress, strain hardening is observed with increased displacement preceding densification of the lattice. Figure 2a shows images extracted from the sample as it deforms. For the open-cell zero-face sample, the vertical struts buckled followed by fracture with force distribution around the unit-cell center. The presence of a single wall results in redistribution of the force with buckling more pronounced opposite to the side with faces. The effect of symmetricity is highlighted in the two-face lattice. When the faces are adjacent to each other (PLA-2-Y), the lack of symmetricity results in pronounced buckling on the side opposite to the faces. When faces are placed symmetrically around the center (PLA-2-O-Y), the sample deforms uniformly. The uniform stress distribution results in an increase in yield stress as well as elongation at break, indicating the key role that non-symmetricity plays in impairing mechanical performance. The values of yield stress are provided in Table 3 and compared in Figure 3a.

The yield stress for the unit cells shows an increase in stress with faces except for the one-face sample whose average shows a decrease. The highest value of yield stress is obtained for PLA-2-O-Y representing a 130% increase over the zero-face (PLA-0-Y). Cognizant of the fact that increasing faces will increase yield stress with a concomitant increase in weight, we review specific stress. The values of specific stress are shown in Table 3. When density is accounted for, the gains in yield stress obtained in faces are realized only for PLA-2-O-Y and PLA-4-Y, which are the two symmetric lattices. Where the maximum improvement (PLA-2-O-Y) in yield stress was 130%, the maximum in specific yield stress was 51%.

The slope of the linear elastic region was analyzed to determine the modulus. The trends of peak stress are generally replicated in modulus values (Figure 3b). For the single-face sample, the modulus shows an increase of 125%. The unsymmetric two-face sample PLA-2-Y shows a 126% increase over the zero-face, while the symmetric two-face PLA-2-O-Y shows a 223% increase. The specific moduli trends are similar with a maximum gain in specific modulus being for PLA-2-O-Y of 47% over that of the zero-face PLA-0-Y lattice.

Next, we examined the unit cells in orthogonal directions (X and Z). In general, all samples in orthogonal directions exhibited a higher modulus and peak stress over the number of face counterparts in the Y direction. First, we looked at the one-face sample. For the same number of faces, the PLA-1-Y showed an increase of 125% over the zero-face, but PLA-1-X exhibited a marked increase of 227%, and PLA-1-Z had a 146% increase over the zero-face unit cell. This indicates that changing orientation resulted in a 138% (4.67 MPa) improvement in PLA-1-X over PLA-1-Y (1.96 MPa) for the max stress. The images captured highlight how face orientation in non-symmetric one-face unit cells results in changes to stress distribution, with the buckling of the SC frame and fracture. Specific yield stress reflects similar trends as that of yield stress. For the unit cell, the maximum gain in yield stress for all orthogonal xz orientations is the PLA-2-O-Z with a 313% and 154% improvement in yield stress and specific yield stress, respectively, over the open-cell PLA-0-Y lattice.

The corresponding moduli increase is 227% for PLA-1-X and 146% for PLA-1-Z over that of the PLA-1-Y. For the two-face, the effect of the two faces placed adjacent to each other shows a 163.5% peak stress, while when the two faces are symmetric around the cube center, a 313% improvement in peak stress is obtained over the zero-face. These reflect a ~90% increase over their Y-direction counterpart. The moduli trends reflect a 200% in the PLA-2-X and a 393% increase in PLA-2-O-Z in moduli over the open-cell unit cell, which is a 50% improvement over their Y-direction counterpart. The three-face lattice trends replicate that of the one-wall lattice with significantly higher values, close to double in peak stress and 135% in modulus. The highest modulus and specific modulus obtained is in PLA-2-O-Z, indicating a 393% and 120% increase, respectively. The full-face lattice indicates improvement through orientations that inhibit the buckling of the struts. An increase in peak stress of 234% (7.15 MPa) and modulus of 321% (174 MPa) in PLA-4-X is obtained over the corresponding zero-face values, which reflects a ~70% increase over the Y counterpart. The unit-cell stress–strain results thus reflect that faces placed to buttress the vertical struts serve to improve modulus and stress.

The 4 × 4 × 4 lattice stress–strain curves are shown in Figure 1b. The curves exhibit changes compared to their single unit-cell counterparts. In general, the stress–strain curves depict an initial linear behavior, a peak indicating some damage, followed by oscillations reflecting progressive damage of different ligaments of the structure. The strut-based zero-face lattice shows no change in shape in the 4 × 4 × 4 compared to the unit cell. The initial damage experienced at the peak leads to no sustained load-bearing capability at higher elongations. When one face is placed in the Y direction, the stress–strain curve mirrors that of the zero-face. The two- and three-face mirror this with increased fluctuations with higher elongations. Orthogonal directions show no improvement in placing one face in the z or x direction in an extended load-bearing capacity. PLA-2-X and PLA-2-O-X, however, demonstrate extended elastic–plastic behavior past the peak value without an initial strain softening. This shape is also replicated in the PLA-4-Y lattice. The orthogonal Z-direction all-face lattice PLA-4-Z shows the highest peak stress with strain softening and elastic–plastic behavior.

Compared to the unit cell, the zero-face 4 × 4 × 4 sample shows a drop in peak stress of 6% and an increase in modulus of 142%. Moduli are uniformly improved for all lattices. The scaled-up lattices show increased modulus over that of the 4 × 4 × 4 zero-face lattice as well as over their unit-cell counterparts. The time-lapse images for the scaled-up samples (Figure 2b) reflect the propagating nature of the fracture initiation arising from unsymmetric loading, stress concentration, and inhibition of buckling. One can infer that the strut lattice exhibits limited ability for damage tolerance, with fracture following swiftly once peak stress has been reached. The all-face lattice shows significant improvement in damage tolerance. The orientation of the faces with respect to the loading directions in the four-face lattice reflects the better transfer of stress in the plateau region when the faces are oriented around the cube center symmetric to the loading direction. When the faces buttress the vertical struts, a higher peak load is experienced but in the plateau region, where the higher oscillations reflect a higher propensity for localized fractures across the sample. It is to be noted that the absence of these oscillations in the unit cell for all lattices reflects that the stress transfer transition from multiple localized events to uniform resistance with low oscillations is a key aspect to be considered when scaling up. 

As the table and graphs indicate, the mechanical properties determined from the simulations show a deviation from the experimental results in magnitude with the retention of trends. The deviation from the simulation is attributed to two issues. First, the low resolution of FDM 3D printing is inherent to the process of layer-by-layer deposition of melt followed by solidification concurrent to deposition of subsequent layers. Secondly, higher manufacturing defects occur from the layer-stepping effect, leading to stress concentration points within structures. Abbot et al. [31] explored this experimental and FEA investigation of samples printed with a range of infills, i.e., the ratio of solid material to empty space inside a model, usually expressed as a percentage. They observed that even though the FEA had some agreement with the samples printed with up to 50% infill, it varied greatly from the samples printed at higher infills primarily because of the increased number of defects in the samples with increased density and layers. As our samples have a strut diameter and wall thickness of 1.5 mm, they were printed at 100% infill. This increases the dominance of inter-layer defects on the failure of the samples under compression and contributes more toward non-agreement with the FEM. The increased number of defects in FDM-printed PLA and catastrophic failures under compression was also reported by Mishra et al. [32] However, a consistent decrease in simulation moduli versus the experimental was also evident. Most notably, unlike the experimental results where the scale-up of the unit cell is reflected by an increase in magnitudes, the simulations show a decrease. We attribute the effect to the use of compression stress–strain data for the simulations, which reflect a brittle polymer with low strain to failure compared to the evidence in the experimental frames extracted from the test. In analyzing in-plane and out-of-plane deformation of honeycombs, Gibson [1] notes that for deformation in struts that are angled, both flexure and axial deformation play a role. Even with the limits on magnitude, the FEA results provide a mechanism for the enhancement of performance. Figure 3c provides images for both the single and 4 × 4 × 4 frames. The stress contours of all the lattices show the stress concentration clearly visible at the nodes of the lattice types for which the faces do not support the vertical columns. The failure occurs for those lattices at the nodes, while for the lattices for which the faces directly reinforce the vertical struts, uniform and gradual dissipation of the stress is observed, resulting in the higher performance of those lattice types. The PLA-3-Z and PLA-2-O-Z are key lattices that have all four vertical struts reinforced by the faces and demonstrate the best lightweighting properties, both numerically and experimentally. We note that the zero-wall strut-only frame lattice shows stress concentrations at the nodes and higher stress in the vertical members. As plates are inserted, the vertical member bears lower stress. In the scale-up comparison, we note that buckling present in the unit cell is impeded in the 4 × 4 × 4 lattice when plates are present, particularly when the plate is able to buttress the vertical strut. We extracted three units to highlight key design elements emerging. In Figure 3d, the unit cells are subjected to 0.1 mm displacement strain, and the stress contour plots are shown. The lattices PLA-2-O-X, PLA-3-Z, and PLA-4-X feature closed faces that serve to directly reinforce the vertical load-bearing struts. Analysis of the contour plots reveals a redistribution of stress from the nodes to the closed faces, consequently reducing stress concentration at the nodes. Moreover, the vertical closed faces effectively mitigate failure due to the buckling of the vertical struts by transferring the load to the stretching of the horizontal columns. This transfer of load is clearly evidenced by the color transition along the horizontal direction in the contour plots.

### 3.2. Energy Effects

As indicated from the stress–strain curves, both the unit cells and the 4 × 4 × 4 scaled-up lattice for the 13 configurations demonstrated changes in peak stress and elongation. This reflects a change in energy absorption arising from transitions in resistance to buckling in the unit cells through symmetric and unsymmetrical load distribution and stress transfer across the unity cells in the 4 × 4 × 4 lattice. Energy absorption (EA) of the structure during compression can be considered by integration of the stress–strain curve:

$$EA=\int_{0}^{\varepsilon }(\sigma \varepsilon )d\varepsilon$$

**Figure 4:**
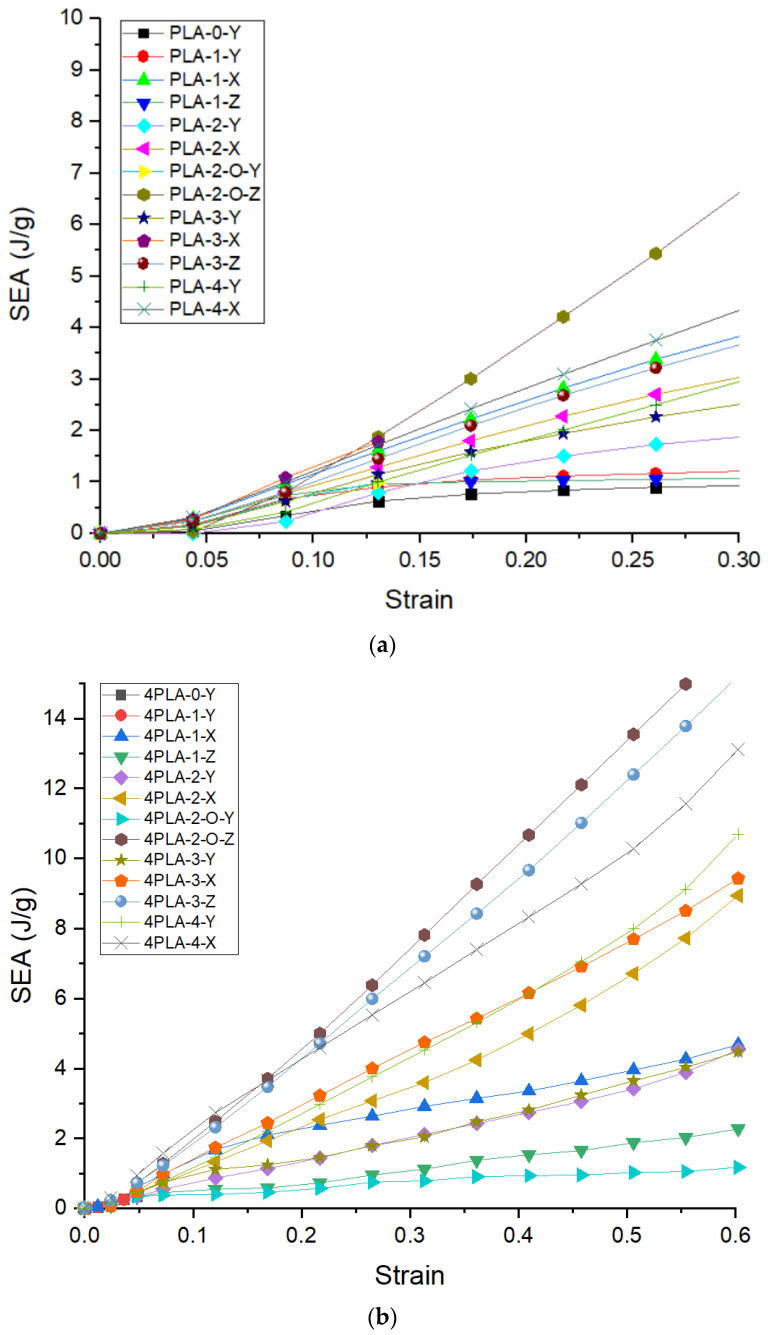
(**a**) SEA of the unit lattice; (**b**) SEA for the 4 × 4 × 4 lattice.

The stress–strain curves indicate linear elastic, strain softening and/or strain hardening, and then densification as the lattice collapses and bulk polymer response ensues. For comparative purposes, Table 3 and Figure 4a,b provide the truncated EA and *SEA* for the unit cell up to 15% strain and 4.5% strain for the 4 × 4 × 4. aSince some of the samples tend to undergo catastrophic failure at the mentioned strains, so to have a fair comparison for all the samples, the area of the stress–strain curves were considered till that point. The trends in EA differ from that of the yield stress, indicating changes in lattice displacement and strain. In the unit cell, the highest EA is obtained in the PLA-4-X and PLA-2-O-Z with 0.93 and 0.88 MJ/m^3^, respectively. This represents a respective improvement of 395% and 368% over that of the open-cell zero-face lattice with 0.19 MJ/m^3^. The scaled-up zero-face lattice shows a drop in EA of 68% to 0.06 MJ/m^3^ in the 4 × 4 × 4 lattice over its single cell, supporting our guiding hypothesis that FDM open lattices have stress concentration at the nodes and low performance in the scaled-up structures. The highest EA A in the 4 × 4 × 4 lattice is obtained in PLA-4-X, indicating that for the scaled-up FDM lattice, the all-face lattice does mitigate challenges presented by stress concentration in FDM at the nodes joining the struts. Comparing the EA in the 4 × 4 × 4 lattice to its single-cell counterpart, we discern that PLA-0-Y and PLA-1-Y indicate losses in scale-up, while all other lattice increase in EA. PLA-2-0-Z indicates the highest improvement in EA going from 1.95 in the unit cell to 5.5 in the 4 × 4 × 4 lattice. Since lattice faces affect density, specific energy absorption was employed, where $\rho_{L}$ is the lattice density

$$SEA=\frac{\int_{0}^{\varepsilon }(\sigma \varepsilon )d\varepsilon }{\rho_{L}}$$

**Figure 5:**
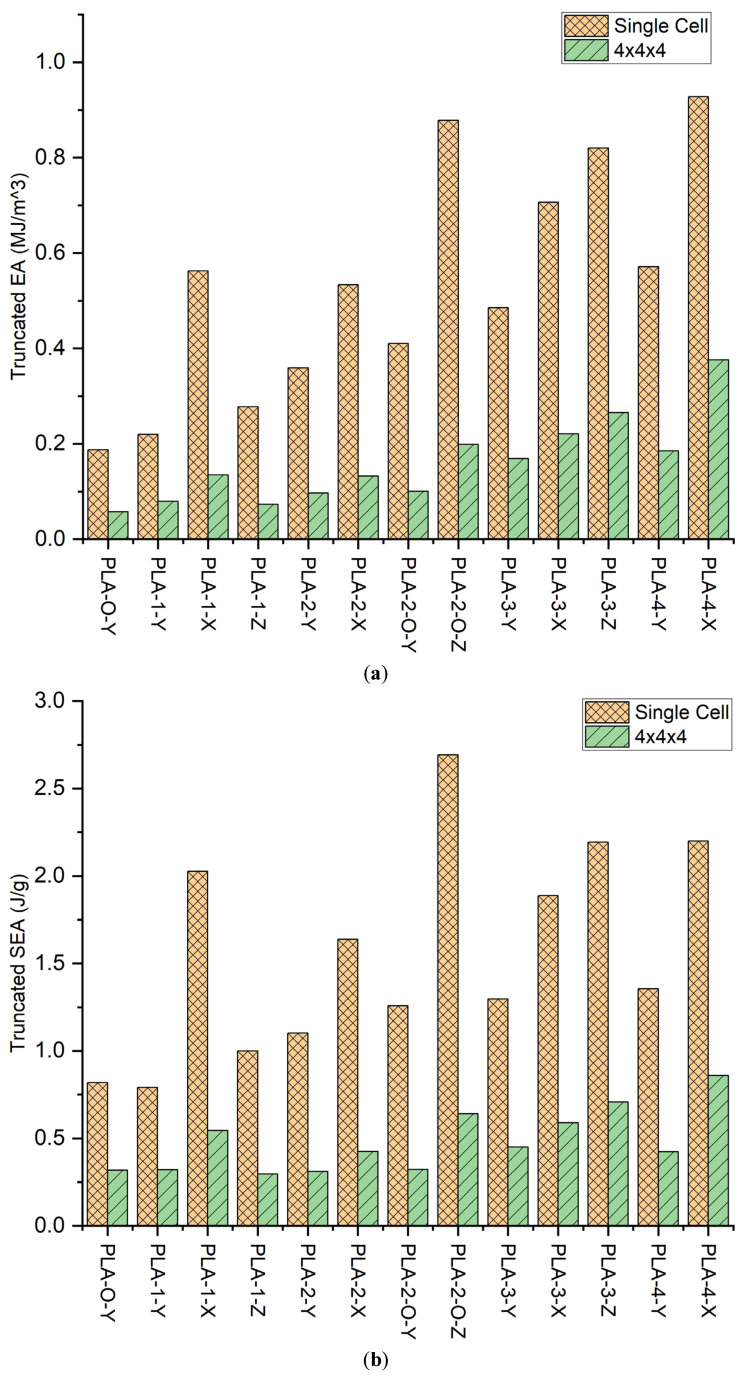
(**a**) Energy absorbed till 15% strain for single cell and 4.5% strain for 4 × 4 × 4; (**b**) Specific energy absorbed till 15% strain for single cell and 4.5% strain for 4 × 4 × 4.

Figure 5a,b show the SEA for the unit cell and 4 × 4 × 4 lattice for the 13 configurations. As can be seen for both the unit cell and the 4 × 4 × 4 lattice, the trends are the same. Including the lattice density, the highest SEA is obtained in PLA-2-O-Z for the unit cell rising from 0.82 J/gm to 2.69 J/gm. Scaling up to the 4 × 4 × 4 lattice has a substantially positive impact on the SEA. A greater than 1000% increase is obtained in nearly all lattices. Which is far higher than the reported 31% increase in the modified FCC lattice using struts [33]. In another study, double gyroid (DG) structures were SLM-printed and subjected to compressive loads and showed ~300% improvement in energy absorption over a BCC-SC lattice structure of comparable relative density [34]. This reflects the positive contribution the stress oscillations have on the energy absorbed. The best EA is obtained in the PLA-2-O-Z sample with a magnitude of 42.36 J/g over its unit-cell counterpart as it is the only lattice type that could reach complete densification without having a catastrophic failure, thus implying minimal structural imperfections. The increase in SEA and SEA is indicative of strain-hardening behavior [35] due to the addition of the faces, which is most profound in PLA-2-O-Z. This is because each face added to the lattice directly reinforces the vertical struts, thus using the full capability of the added material due to the faces.

### 3.3. Transformation in Deformation Mechanism from Addition of Plates

**Figure 6:**
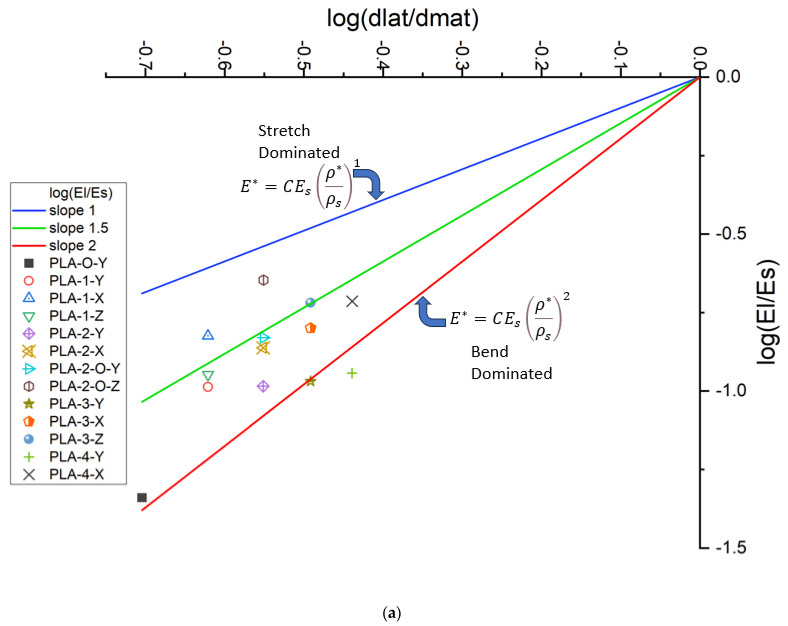

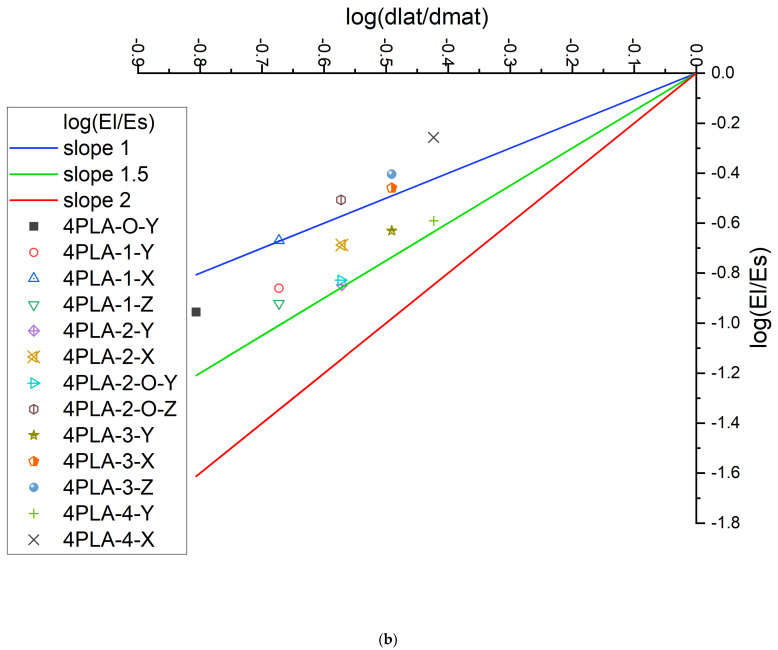
(**a**) Gibson–Ashby plot of PLA unit cells and 4 × 4 × 4 PLA samples; (**b**) Ashby plot for scaled-up PLA for 4 × 4 × 4.

Figure 6a,b show the Ashby–Gibson modulus–density ratios on a log scale for the unit and scaled-up lattice. As there are inherently high defects in the FDM process, which results in catastrophic failure at the yield point, only the elastic region of the data is reliable for comparison on the Ashby–Gibson plot. When the lattice is closer to the slope of one, it is stretch-dominated, and when closer to the slope of two, it is bend-dominated. Pure-stretching lattices have up to three times higher lightweighting compared to pure bending, as established before. The introduction of plates resulted in a migration to increased stretching deformation. The axial orientation of the plates affected the deformation mechanism. For the unit cell, when the plates are fanning across the strut transverse to the loading direction, buckling is inhibited and the stretching is enhanced. Lattice PLA-2-O-Z, being the closest to the line, has slope 1 showing the most stretch-dominated behavior. The lattice is symmetric about the center of mass. Faces directly reinforce the vertical struts resulting in inhibition to buckling. Figure 6b explores the stress transfer across cells in the scaled-up lattice. All values move toward enhanced stretching. Again, trends rather than explicit values are of value. This is because Ashby–Gibson assumes loading with strut and buckling resistance, and the plates with 45-degree placements introduce shear and normal responses to the compressive force.

## 4. Conclusions

The insertion of faces into completely open frames has been shown to be effective in mitigating the stress concentration and scale-up challenges of FDM polymer lattices. A combined strut-face FDM lattice series was explored using sequential insertion of faces into an open BCC-SC lattice. A unit cell and a 4 × 4 × 4 lattice were compressed in the three orthogonal directions. The results indicate that the unit cell showed buckling-dominated failure. When plates are introduced, the vertical strut that is buttressed by the face inhibits buckling and shows improved performance. For the unit cell, the all-face lattice demonstrated advantages over the open lattice with 322%, 234%, and 395% increases in modulus, yield stress, and energy absorption over the zero-face lattice, respectively. Stress concentration at the nodes decreases, as is evident in the FEA images. Examining the effect of additional cells as a means of inhibiting the buckling of a unit cell revealed significant benefits of adding faces. Scaling up the lattice to 4 × 4 × 4 resulted in stress oscillations with deformation associated with buckling and stress transfer. The scale-up showed an increase in the modulus over the unit cell in the range of 6 to 186%. Yield stress remained very similar to that of the single unit cell. Faces were effective in increasing the strain to failure, which was reflected in the energy absorption analysis. The energy absorption for the scaled-up open cell (PLA-0-Y) showed lower performance compared to that of the single cell as the manufacturing defects due to FDM amplified and dominated the failure by not allowing the proper transfer of stress to the following layer of lattices, further supporting the hypothesis of the paper in adding faces. With the addition of the faces, the scaled-up samples showed improvement in the dissipation of the stress, resulting in higher energy absorption, especially in the case of PLA-2-O-Z. This is because of the increased surface area resulting in a more continuous path for material flow in the FDM print, causing fewer defects, in addition to the increased reinforcement of the vertical struts of the lattice structure. Therefore, the addition of the faces lowered the dominance of the manufacturing defects by giving a more continuous path for material flow for FDM compared to that for slender struts of the open lattice, and provided reinforcement for the vertical struts to distribute the stress more uniformly across repeating cells and, hence, reduce the stress concentration. An all-face lattice mitigated this challenge. The trends in mechanical properties with the introduction of faces were dependent on the orientation of the faces and their number. The two-face lattice enabled the identification of a key factor in performance—the symmetric placement of faces. PLA-2-O-Z outperformed all other lattices. In the unit-cell Y direction, moving the faces from adjacent (PLA-2-Y) to symmetric placement (PLA-2-O-Y) resulted in 66% and 43% improvement in yield stress and modulus, respectively. The scaled-up lattice performance reflected advantages for the complete-face lattice with an improvement of 186%, 66%, and 235% in modulus, yield stress, and EA over the single-face counterpart, respectively, for PLA-4-X. Accounting for the density increase from the addition of faces, however, the PLA-2-0-Z and PLA-3-Z provided significant improvement in mechanical performance for the increased weight from the insertion of faces. The results indicate a new design approach using a partially open lattice whose struts are constrained by faces to improve modulus, yield stress, and energy absorption for lightweight structures, which has never been undertaken before. 

## Figures and Tables

**Table 1:** Unit cells of the lattice.

Lattice Types	Center of Mass	X Orientation	Y Orientation	Z Orientation
PLA-0	(0, 0, 0)	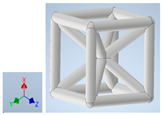	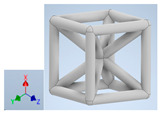	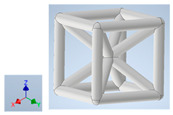
PLA-1	(0.58856, 0, 0)	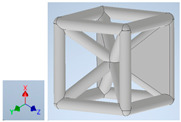	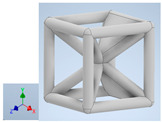	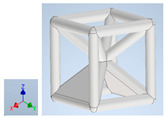
PLA-2	(0.50247, 0, −0.50224)	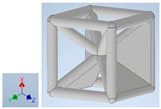	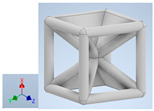	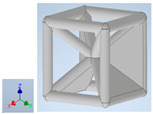
PLA-2-O	(0, 0, 0)	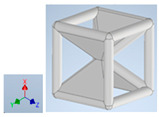	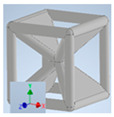	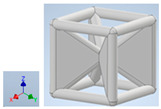
PLA-3	(0, 0, −0.43692)	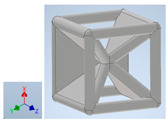	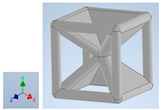	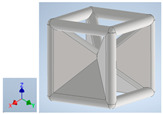
PLA-4	(0, 0, 0)	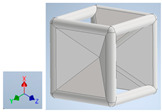	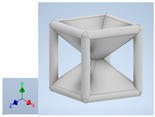	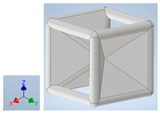

**Table 2:** Bulk Material properties acquired.

	Modulus (MPa)	Yield Point (MPa)	Strain to Failure
Flexural Modulus	2031.69 ± 39.35	43.60 ± 8.07	1.54%
Tensile tests	1036.83 ± 35.69	34.84 ± 2.69	4.50%
Compression tests	903.84 ± 61.33	56.60 ± 2.99	No Failure

**Table 3:** Results of the modulus, stress, and energy for all lattices.

Lattice Types	Mass Moment of Inertia (kg.m^2^)	Density-Lattice (kg/m^3^)	Modulus (MPa)	% Change in Modulus with Respect to Open Cell	Simulation Modulus (Mpa)	Sp. Modulus Mpa-m^3^/kg	% Change in Sp. Modulus with Respect to Open Cell	Yield Strength (MPa)	% Change in Yield Strength with Respect to Open Cell	Sp. Yield Strength*10^3^ Mpa-m^3^/kg	% Change in Sp. Yield Strength with Respect to Open Cell	Truncated EA (MJ/m^3^)	% Change in Truncated EA Strength with Respect to Open Cell	Truncated SEA (J/g)	% Change in Truncated SEA Strength with Respect to Open Cell	EA (MJ/m^3^)	% Change in EA Strength with Respect to Open Cell	SEA (J/g)	% Change in SEA Strength with Respect to Open Cell
**PLA-O-Y**	9.70 × 10^−09^	229.12	41.34 ± 10.42	0.00	62.25	0.29	0.00	2.14 ± 0.39	0.00	9.20	0.00	0.19	0.00	0.82	0.00	0.80	0.00	3.48	0.00
**PLA-1-Y**	1.05 × 10^−08^	277.67	93.10 ± 7.22	125.21	73.31	0.29	0.18	1.96 ± 0.41	−8.41	12.40	34.78	0.22	17.12	0.79	−3.36	0.97	21.25	3.49	0.05
**PLA-1-X**	1.12 × 10^−08^	277.67	135.35 ± 6.47	227.41	106.32	0.51	76.46	4.67 ± 0.52	118.22	16.38	77.99	0.56	200.05	2.03	147.59	1.08	35.00	3.89	11.40
**PLA-1-Z**	1.06 × 10^−08^	277.67	101.88 ± 8.63	146.44	63.41	0.37	28.43	3.68 ± 0.17	71.96	13.26	44.13	0.28	48.01	1	22.13	0.48	−40.00	1.73	−50.49
**PLA-2-Y**	1.14 × 10^−08^	325.89	93.53 ± 11.65	126.25	83.00	0.32	12.72	2.97 ± 0.39	38.79	13.18	43.24	0.36	91.54	1.1	34.67	1.22	52.50	3.74	7.22
**PLA-2-X**	1.21 × 10^−08^	325.89	124.10 ± 37.52	200.19	118.99	0.50	73.58	5.64 ± 0.07	163.55	13.00	41.35	0.53	184.63	1.64	100.12	1.14	42.50	3.49	0.19
**PLA-2-O-Y**	1.16 × 10^−08^	326.22	133.66 ± 1.73	223.32	72.78	0.42	47.02	4.93 ± 0.22	130.37	13.92	51.29	0.41	118.80	1.26	53.67	0.39	−51.25	1.19	−65.76
**PLA-2-O-Z**	1.29 × 10^−08^	326.22	203.92 ± 5.51	393.28	113.34	0.63	120.38	8.84 ± 0.25	313.08	23.35	153.78	0.88	368.25	2.69	228.88	13.82	1627.50	42.37	1113.32
**PLA-3-Y**	1.25 × 10^−08^	374.10	97.00 ± 12.60	134.64	99.93	0.27	−5.90	3.34 ± 0.40	56.07	13.08	42.23	0.49	158.92	1.3	58.58	1.88	135.00	5.01	43.93
**PLA-3-X**	1.30 × 10^−08^	374.10	143.48 ± 6.78	247.07	108.33	0.47	64.64	7.19 ± 0.31	235.98	11.77	27.97	0.71	276.63	1.89	130.67	0.90	12.50	2.40	−31.10
**PLA-3-Z**	1.38 × 10^−08^	374.10	172.63 ± 9.04	317.59	158.19	0.50	73.25	7.40 ± 0.22	245.79	19.78	115.00	0.82	337.50	2.19	167.95	1.49	86.25	3.98	14.07
**PLA-4-Y**	1.35 × 10^−08^	421.99	103.00 ±34.88	149.15	117.09	0.30	5.79	4.53 ± 0.40	111.68	14.55	58.17	0.57	204.76	1.35	65.47	2.83	253.75	6.71	92.07
**PLA-4-X**	1.47 × 10^−08^	421.99	174.37 ± 34.85	321.79	194.09	0.46	60.55	7.15 ± 0.62	234.11	16.02	74.12	0.93	395.01	2.2	168.77	1.72	115.00	4.07	16.74
**4PLA-O-Y**	3.58 × 10^−06^	181.20	100.13 ± 1.80	0.00	41.12	0.55	0.00	1.98 ± 0.06	0.00	10.92	0.00	0.06	0.00	0.32	0.00	0.06	0.00	0.35	0.00
**4PLA-1-Y**	4.69 × 10^−06^	246.45	124.41 ± 0.36	24.25	47.29	0.50	−8.65	2.72 ± 0.06	37.75	11.06	1.28	0.08	37.54	0.32	1.12	0.07	16.67	0.30	−14.22
**4PLA-1-X**	4.73 × 10^−06^	246.45	193.50 ± 3.13	93.25	79.38	0.79	42.08	4.02 ± 0.04	103.04	16.30	49.28	0.13	133.49	0.55	71.67	1.23	1950.00	5.00	1407.22
**4PLA-1-Z**	4.70 × 10^−06^	246.45	108.42 ± 2.58	8.28	71.51	0.44	−20.39	2.63 ± 0.02	33.06	10.68	−2.17	0.07	26.33	0.3	−7.12	0.65	983.33	2.64	696.50
**4PLA-2-Y**	5.79 × 10^−06^	311.26	128.29 ± 3.92	28.12	59.70	0.41	−25.41	2.85 ± 0.17	44.26	9.17	−16.02	0.10	67.81	0.31	−2.31	1.56	2500.00	5.03	1413.56
**4PLA-2-X**	5.85 × 10^−06^	311.26	185.54 ± 11.42	85.30	91.13	0.60	7.87	3.66 ± 0.14	85.03	11.76	7.71	0.13	129.89	0.43	33.83	3.04	4966.00	9.77	2849.50
**4PLA-2-O-Y**	5.80 × 10^−06^	310.25	134.25 ± 4.65	34.08	83.98	0.43	−21.69	3.41 ± 0.06	72.43	10.99	0.71	0.10	74.10	0.32	1.68	0.52	766.00	1.66	406.17
**4PLA-2-O-Z**	5.88 × 10^−06^	310.25	281.27 ± 9.64	180.90	126.73	0.91	64.06	7.60 ± 0.03	284.18	24.49	124.38	0.20	245.08	0.64	101.54	5.54	9133.00	17.85	5292.61
**4PLA-3-Y**	5.80 × 10^−06^	374.63	211.60 ± 5.96	111.33	77.96	0.56	2.21	5.43 ± 0.17	174.41	14.49	32.72	0.17	193.03	0.45	41.73	0.78	1200.00	2.09	528.78
**4PLA-3-X**	5.80 × 10^−06^	374.63	313.98 ± 13.32	213.57	125.34	0.84	51.67	8.25 ± 0.23	316.92	22.01	101.66	0.22	283.95	0.59	85.71	3.91	6416.67	10.44	3051.95
**4PLA-3-Z**	5.88 × 10^−06^	374.63	356.72 ± 14.32	256.26	141.03	0.95	72.31	8.26 ± 0.07	317.71	22.06	102.03	0.27	360.73	0.71	122.84	5.88	9700.00	15.68	4640.02
**4PLA-4-Y**	9.70 × 10^−06^	437.55	231.85 ± 24.11	131.55	100.32	0.53	−4.11	5.41 ± 0.52	173.27	12.35	13.17	0.19	221.56	0.42	33.16	5.02	8266.67	11.48	3364.79
**4PLA-4-X**	1.00 × 10^−05^	437.55	500.11 ± 8.99	399.46	199.70	1.14	106.84	11.87 ± 0.25	500.26	27.14	148.58	0.38	553.08	0.86	170.45	5.77	9516.67	13.18	3882.44

## Data Availability

The original contributions presented in the study are included in the article, further inquiries can be directed to the corresponding author.

## References

[1] Gibson L.J., Ashby M.F. (1997). Cellular Solids: Structure and Properties.

[2] Lim J.-H., Kang K.-J. (2006). Mechanical behavior of sandwich panels with tetrahedral and Kagome truss cores fabricated from wires. Int. J. Solids Struct..

[3] Bonatti C., Mohr D. (2017). Large deformation response of additively-manufactured FCC metamaterials: From octet truss lattices towards continuous shell mesostructures. Int. J. Plast..

[4] Maconachie T., Leary M., Lozanovski B., Zhang X., Qian M., Faruque O., Brandt M. (2019). SLM lattice structures: Properties, performance applications and challenges. Mater. Des..

[5] Tancogne-Dejean T., Diamantopoulou M., Gorji M.B., Bonatti C., Mohr D. (2018). 3D Plate-Lattices: An Emerging Class of Low-Density Metamaterial Exhibiting Optimal Isotropic Stiffness. Adv. Mater..

[6] Bitzer T. (1997). Honeycomb Technology.

[7] Oluwabunmi K., D’souza N.A., Zhao W., Choi T.-Y., Theyson T. (2020). Compostable, fully biobased foams using PLA and micro cellulose for zero energy buildings. Sci. Rep..

[8] Ashby M.F. (2006). The properties of foams and lattices. Philos. Trans. R. Soc. A Math. Phys. Eng. Sci..

[9] Deshpande V.S., Fleck N.A., Ashby M.F. (2001). Effective properties of the octet-truss lattice material. J. Mech. Phys. Solids.

[10] Pellegrino S., Calladine C. (1986). Matrix analysis of statically and kinematically indeterminate frameworks. Int. J. Solids Struct..

[11] Deshpande V.S., Ashby M.F., Fleck N.A. (2001). Foam topology: Bending versus stretching dominated architectures. Acta Mater..

[12] Meza L.R., Das S., Greer J.R. (2014). Strong, lightweight, and recoverable three-dimensional ceramic nanolattices. Science.

[13] Evans A. (2001). Lightweight Materials and Structures. MRS Bull..

[14] Tancogne-Dejean T., Mohr D. (2018). Elastically-isotropic elementary cubic lattices composed of tailored hollow beams. Extrem. Mech. Lett..

[15] Xu S., Shen J., Zhou S., Huang X., Xie Y.M. (2016). Design of lattice structures with controlled anisotropy. Mater. Des..

[16] Messner M.C. (2016). Optimal lattice-structured materials. J. Mech. Phys. Solids.

[17] Tancogne-Dejean T., Mohr D. (2018). Elastically-isotropic truss lattice materials of reduced plastic anisotropy. Int. J. Solids Struct..

[18] Gurtner G., Durand M. (2014). Stiffest elastic networks. Proc. R. Soc. A Math. Phys. Eng. Sci..

[19] Liu Z., Zhao R., Tao C., Wang Y., Liang X. (2023). Mechanical Performance of a Node-Reinforced Body-Centered Cubic Lattice Structure: An Equal-Strength Concept Design. Aerospace.

[20] Wang P., Yang F., Zhao J. (2022). Compression behaviors and mechanical properties of modified face-centered cubic lattice structures under quasi-static and high-speed loading. Materials.

[21] Li B., Shen C. (2022). Solid Stress-Distribution-Oriented Design and Topology Optimization of 3D-Printed Heterogeneous Lattice Structures with Light Weight and High Specific Rigidity. Polymers.

[22] Lohmuller P., Favre J., Piotrowski B., Kenzari S., Laheurte P. (2018). Stress Concentration and Mechanical Strength of Cubic Lattice Architectures. Materials.

[23] Boniotti L., Foletti S., Beretta S., Patriarca L. (2020). Analysis of strain and stress concentrations in micro-lattice structures manufactured by SLM. Rapid Prototyp. J..

[24] Berger J.B., Wadley H.N.G., McMeeking R.M. (2017). Mechanical metamaterials at the theoretical limit of isotropic elastic stiffness. Nature.

[25] Medori E. (2021). Mechanical Behavior of FDM Printed Lattice Structures with Potential for Biomedical Application. Master’s Thesis.

[26] (2014). Standard Test Method for Tensile Properties of Plastics.

[27] (2015). Standard Test Method for Compressive Properties of Rigid Plastics.

[28] (1997). Standard Test Methods for Flexural Properties of Unreinforced and Reinforced Plastics and Electrical Insulating Materials. Annual book of ASTM Standards.

[29] Righetti M.C., Gazzano M., Di Lorenzo M.L., Androsch R. (2015). Enthalpy of melting of α′- and α-crystals of poly(l-lactic acid). Eur. Polym. J..

[30] Vakharia V.S., Kuentz L., Salem A., Halbig M.C., Salem J.A., Singh M. (2021). Additive Manufacturing and Characterization of Metal Particulate Reinforced Polylactic Acid (PLA) Polymer Composites. Polymers.

[31] Abbot D., Kallon D., Anghel C., Dube P. (2019). Finite Element Analysis of 3D Printed Model via Compression Tests. Procedia Manuf..

[32] Mishra A.K., Chavan H., Kumar A. (2021). Effect of material variation on the uniaxial compression behavior of FDM manufactured polymeric TPMS lattice materials. Mater. Today Proc..

[33] Wang P., Yang F., Li P., Zheng B., Fan H. (2021). Design and additive manufacturing of a modified face-centered cubic lattice with enhanced energy absorption capability. Extrem. Mech. Lett..

[34] Maskery I., Aboulkhair N., Aremu A., Tuck C., Ashcroft I. (2017). Compressive failure modes and energy absorption in additively manufactured double gyroid lattices. Addit. Manuf..

[35] Li Q.M., Magkiriadis I., Harrigan J.J. (2006). Compressive strain at the onset of densification of cellular solids. J. Cell. Plast..

